# Preparation of Rat Gingival Mitochondria with an Improved Isolation Method

**DOI:** 10.1155/2010/275103

**Published:** 2010-11-21

**Authors:** Noriaki Kaneko, Tetsuya Rikimaru, Tetsuyuki Fujimura, Shigeyasu Mori, Saburo Hidaka, Hidehiro Kaya

**Affiliations:** ^1^Department of Dental Hygiene, Fukuoka College of Health Sciences, 2-15-1 Tamura, Sawara-Ku, Fukuoka 814-0193, Japan; ^2^Fujimura Clinic, Fukuoka, Japan; ^3^Hamadayama Smile Dental Clinic, Tokyo, Japan

## Abstract

In order to establish a method of obtaining rat gingival mitochondria (Mt), Mt fractions were prepared in various combinations of homogenizing time with collagenase concentration. Rat gingival tissues were excised, minced, treated with collagenase, homogenized, and subjected to differential centrifugation rates. Both the respiratory control ratio (RCR) and adenosine diphosphate/oxygen (ADP/O) ratio of the Mt fraction prepared in a combination of 40, 50, or 60 sec homogenization with collagenase in a concentration range of 0.115%–0.130% (w/v) were measured. The values for the RCR and ADP/O ratio of the Mt fraction obtained in an optimal condition was 1.80 ± 0.05 and 1.65 ± 0.03, respectively. These results suggest that Mt of fairly high quality can be obtained through this refined combination of the homogenizing time and collagenase concentration.

## 1. Introduction

Mitochondria play a central role in the energy metabolism of brain, heart, and muscle by controlling the production of adenosine triphosphate (ATP), and mitochondrial preparations are commonly used to evaluate the metabolic activities of these tissues in both the normal and diseased states [1]. However, oxidative phosphorylation (ATP synthesis) and electron transport in oral tissues, for example, the oral mucosa and gingival tissue, were extensively studied in a period of the late 1970s to early 1980s by several investigators [2–8]. Mitochondria in the oral tissues of animals and humans have been isolated by differential centrifugation of tissue homogenates with or without collagenase treatment [2–5]. Moreover, to avoid the destruction of mitochondria or the loss of respiratory activity, chilled buffer solutions of an isotonic tension have generally been adopted. However, those studies have yielded variable and generally low levels of respiration both in the presence and absence of substrate, and the quantitative assessment of mitochondrial ATP synthesis in oral tissues has been hindered by the extreme difficulty of homogenizing these tissues without damaging mitochondrial integrity and uncoupling oxidative phosphorylation [4]. Therefore, further refinements for the isolation method are still required to obtain mitochondria with high quality. 

Clarification of the role played by oxidative phosphorylation in gingival tissue metabolism would aid in the understanding of responses to local injury and periodontal disease. For example, collagen is a primary constituent of gingival tissue, and its synthesis and remodeling during periodontal tissue repair have been reported to be affected by diabetes [9], scurvy [10], and growth factors [11]. The events which affect these repair activities may also reflect changes at the oxidative phosphorylation level, since ATP is required for many synthetic processes associated with collagen synthesis. 

We report here a procedure for the isolation of mitochondria from rat gingival tissues by an optimized combination between homogenizing time and collagenase concentration, which displayed a good respiratory control ratio (RCR) and adenosine diphosphate/oxygen (ADP/O) ratio using succinic acid as the substrate. Furthermore, the effect on respiratory activity of the leukotoxin (Lx) and lipopolysaccharide (LPS) involved in the inflamed state was examined.

## 2. Materials and Methods

### 2.1. Chemicals

Chemicals used where mannitol, sucrose, potassium chloride (KCl), potassium dihydrogen phosphate (KH_2_PO_4_), succinic acid, rotenone, and adenosine 5^'^-diphosphate sodium salt (ADP) and bovine serum albumin (BSA) were purchased from Sigma-Aldrich Japan Co. (Tokyo, Japan). Collagenase from *Clostridium histolyticum* (Type I), 3-N-morpholinopropanesulfonic acid (MOPS), 4Na-ethylenediaminetetraacetic acid (EDTA), and N-2-hydroxyethylpiperazine-N^'^-2-ethansulfonic acid (HEPES) were purchased from Sigma-Aldrich Co. (St Louis, MO, USA). Leukotoxin (Lx) is a product of Cayman Chemicals Co., USA and lipopolysaccaride (LPS) from *Escherichia coli* is a product of Difco Laboratories, Mich, USA. Other reagents were purchased from ABIOZ Co. Ltd (Osaka, Japan).

### 2.2. Animals

Seven-to-nine week-old male Wistar rats, weighing 250–300 g were purchased from Seac Yoshitomi Ltd (Fukuoka Prefecture). 

### 2.3. Preparation of Gingival Mitochondria

**Figure 1:**
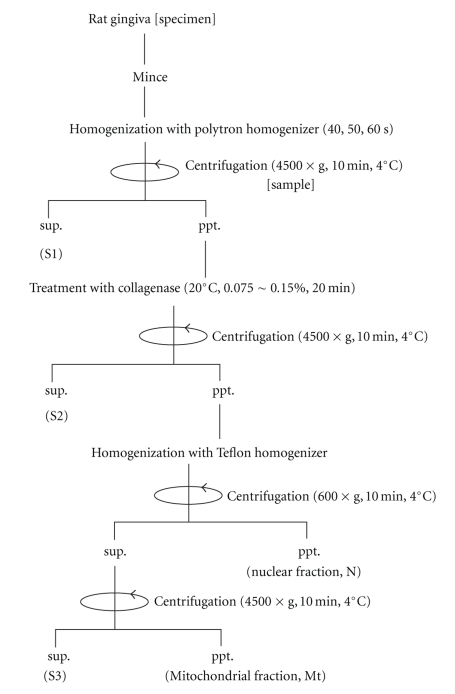
Diagrammatic representation of the mitochondrial (Mt) preparation procedure from gingival tissue in rats. Tissue specimens were washed and minced with 5.0 mM MOPS buffer (pH 7.4) and then intermittently homogenized at 4°C using a polytron homogenizer for 40, 50, and 60 sec. The homogenized gingival sample was centrifuged at 4,500 g for 10 min, the supernatant was labeled as S1, and the sediment was incubated in a Hanks solution (pH 7.4) containing 0.115%–0.130% (w/v) collagenase at 20°C for 20 min. Following the enzymatic treatment, the homogenized gingival sample was centrifuged at 4,500 g for 10 min. When the supernatant is labeled S2, the sediment was diluted with 50 mM MOPS buffer containing 100 mM KCl, 0.2 mM EDTA, and 0.2% (w/v) BSA and homogenized using a Teflon homogenizer for one min. The homogenate was centrifuged at 600 g for 10 min and the sediment was collected as the nuclear (N) fraction. The supernatant was then centrifuged at 4,500 g for 10 min, the supernatant was labeled as S3, and the sediment was finally collected as the mitochondrial (Mt) fraction.

After sacrificing the rats by cervical dislocation under chloroform anesthesia, the gingival tissue specimen (approximately 1.0 g wet weight) was excised from the mandible and maxilla. As shown in Figure 1, tissue specimens were washed and minced with 5.0 mM MOPS buffer (pH 7.4) containing 200 mM mannitol, 70 mM sucrose and 0.05 mM EDTA at 4°C. Minced gingival specimens were diluted 10 fold with 1.2 mM MOPS buffer (pH 7.4) containing 53.9 mM mannitol, 17.2 mM sucrose, 0.05 mM EDTA and 0.1% (w/v) BSA, and then intermittently homogenized at 4°C using a polytron homogenizer (RT 10–35, Kinematic AG, Lucerne, Switzerland) for 40, 50, and 60 sec. The homogenized gingival sample was then centrifuged at 4,500 g for 10 min, the supernatant was labeled as S1 and the sediment was incubated in a Hanks solution (pH 7.4) containing 0.115%–0.130% (w/v) collagenase at 20°C for 20 min. Following the enzymatic treatment, the homogenized gingival sample was centrifuged at 4,500 g for 10 min. While the supernatant was labeled as S2, the sediment was diluted with 50 mM MOPS buffer containing 100 mM KCl, 0.2 mM EDTA, and 0.2% (w/v) BSA and homogenized using a teflon homogenizer for one min. The homogenate was centrifuged at 600 g for 10 min and the sediment was collected as the nuclear (N) fraction. The supernatant was then centrifuged at 4,500 g for 10 min, the supernatant was labeled as S3 and the sediment was finally collected as the mitochondrial (Mt) fraction. 

### 2.4. Specific Activity of Succinate Dehydrogenase (SDH)

The specific activity of succinate dehydrogenase (SDH) was measured by a modified Slater method [12]. The ability of both the supernatant fractions S1, S2, and S3 and the sediment fractions N and Mt to reduce potassium ferricyanide was measured. Protein was determined by the Lowry Folin phenol reagent method [13] using BSA as a standard.

### 2.5. Measurement of the Oxidative Phosphorylation

**Figure 2:**
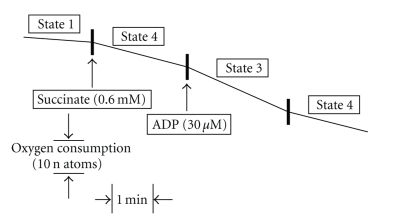
A polarographic trace of gingival mitochondria (Mt). Mt were prepared from approximately 1.0 g rat gingival tissue using an optimized preparative procedure displaying apparent respiratory activity. The preparation conditions are 50 sec of polytron homogenizer treatment and 0.120% (w/v) collagenase. Oxygen consumption by the gingival Mt (0.5 mg protein/ 600 *μ*L buffer) was measured with the addition of substrate (0.6 mM succinate) followed by 30 *μ*M adenosine diphosphate (ADP) at 25°C.

The oxidative phosphorylation of gingival mitochondria was measured using a Biological Oxygen Monitor System (Model 5300: YSI Inc., Ohio, USA). Based on the oxygen consumption of the mitochondrial factions at 25°C, states 1, 3, and 4, that were established by Chance et al. [14], were calculated. Namely, a final concentration of 0.5 mg protein/600 *μ*L buffer of gingival mitochondria was suspended in 600 *μ*L of 10 mM HEPES buffer (pH 7.4) containing 0.25 M sucrose and 10 mM KH_2_PO_4_ in the reaction container of an oxygen electrode apparatus, followed by the addition of 100 *μ*g/mL rotenone and 0.6 mM succinic acid (See Figure 2). After measuring states 1, 3, and 4, the respiration activity of gingival mitochondria was assessed by measuring (1) respiratory control ratio (RCR): the respiratory rate (State 3) in the presence of ADP compared to the rate (State 4) following the expenditure of ADP and (2) the adenosine diphosphate/oxygen (ADP/O) ratio: the ratio of ADP removed from the media to the amount of oxygen consumed, according to the Estabrook method [15].

### 2.6. Effects of Leukotoxin and Lipopolysaccharide on Respiratory Control Ratio (RCR) of Mitochondria

At a concentration range of 0.5–2.0 *μ*g/mg mitochondrial (Mt) protein, leukotoxins (Lxs) were added to a 600 *μ*L of 10 mM HEPES buffer (pH 7.4) containing 0.25 M sucrose and 10 mM KH_2_PO_4_ in the reaction container of an oxygen electrode apparatus, followed by the addition of 100 *μ*g/mL rotenone and 0.6 mM succinic acid. At a concentration range of 5.0–25.0 *μ*g/mg Mt protein, lipopolysaccharide (LPS) was added in the same manner. After measuring states 1, 3, and 4, the respiration activity of gingival mitochondria was assessed by measuring RCR in the same manner as described above.

### 2.7. Ethics

The mitochondrial preparations from rats were carried out at the Fukuoka Dental College, and the study was approved by the Institutional Use and Care of Animal Committee. All procedures were in accordance with the Guidelines on Animal Experiments in Fukuoka Dental College and performed following the Government Law Concerning the Protection and Control of Animals (no. 221) and Japanese Government Notification on Feeding and Safekeeping of Animals (no. 6). 

### 2.8. Statistical Analysis

The data were obtained from 5 times measurements and were expressed as the mean ± standard deviations. Statistical comparisons were made by ANOVA and Scheffe's test using a statistical software program. *P* < .05 was considered significant.

## 3. Results

### 3.1. Succinate Dehydrogense (SDH) Activity in the Different Fractions in the Process of Mitochondrial Isolation

Rat gingival tissues were processed in the course of mitochondrial isolation (Figure 1). The first, second, and third supernatant fractions were designated as S1, S2, and S3, respectively, while the two precipitates were designated as the nuclear (N) and mitochondrial (Mt) fractions. As shown in Table 1, when the three supernatant and two precipitated fractions were obtained in the combination of 50 sec homogenization with 0.120% (w/v) collagenase treatment, the specific activities of succinate dehydrogense (SDH) in the S1, S2, and S3 fractions were 0.51 ± 0.05, 0.72 ± 0.05, and 0.22 ± 0.02, respectively, whereas those of N and Mt precipitated fractions were 0.25 ± 0.02 and 5.67 ± 0.32, respectively.

### 3.2. The Respiratory Control Ratio (RCR) and Adenosine Diphosphate/Oxygen (ADP/O ) Ratio in Mitochondrial Fractions

As shown in Table 2, after 40 sec homogenization with 0.125% (w/v) collagenase treatment of the Mt fraction, the values of the respiratory control ratio (RCR), and adenosine diphosphate/oxygen (ADP/O) ratio were 1.54 ± 0.02 and 1.39 ± 0.05, respectively. After 50 sec homogenization with 0.120% (w/v) collagenase treatment of the Mt fraction, the RCR and ADP/O values were 1.80 ± 0.05 and 1.65 ± 0.03, respectively. Further, after homogenization for 60 sec with 0.120% (w/v) collagenase treatment of Mt fraction, the Mt fraction RCR and ADP/O values were 1.57 ± 0.03 and 1.49 ± 0.01, respectively. The values of the Mt fraction in the 50 sec homogenization with 0.120% (w/w) collagenase treatment were significantly the highest. 

### 3.3. Effects of Leukotoxin and Lipopolysaccharide on Respiratory Control Ratio (RCR) of Mitochondrial Fractions

As shown in Table 3, at a concentration range of 0.5–2.0 *μ*g/mg Mt protein, leukotoxin (Lx) decreased the RCR of the Mt fractions by 29%, 37%, and 42% respectively, compared to the control (None). Furthermore, at a concentration range of 5.0–25.0 *μ*g/mg Mt protein, lipopolysaccharide (LPS) decreased the RCR of the Mt fractions by 31%, 38%, and 40% respectively, compared to the control (none).

## 4. Discussion

There are a few reports in the literature on the preparation of mitochondria (Mt) from epithelial tissue for the purpose of the direct measurement of respiratory activity [2–7, 16, 17]. However, neither the damage to fibroblasts in Mt disease [16] nor the effect of nicotine on Mt in periodontal tissues [17] has ever been reportedly investigated by the measurement of respiratory activity. Moreover, Rosett et al. [2] have suggested that it is impossible to prepare Mt from attached gingival tissue, because of the presence of collagen, which disturbs the isolation of Mt. In the present study of gingival Mt preparation, the changes of the duration of the homogenization time and the concentration of collagenase were investigated. Succinic acid dehydrogenase (SDH) is an enzyme localized within cells in the Mt matrix and an important step in the Krebs cycle [13, 17]. The specific activity of SDH from the Mt fraction was higher by 7.8–25.8 times in the three supernatants, S1, S2, and S3, and compared with the one precipitated in the N fractions (Table 1). This indicates that Mt were obtained through this isolation method that is a combination of a certain specific homogenization time and collagenase concentration. The combined conditions of 50 sec homogenization with 0.120% (w/v) collagenase concentration yielded 1.80 ± 0.05 as the respiratory control ratio (RCR) and 1.65 ± 0.03 as the adenosine diphosphate/oxygen ratio (ADP/O). Fine et al. [4] reported that the RCR and ADP/O ratio from a crude Mt preparation of weanling rat attached gingiva for succinate were 4.0 ± 0.2 and 1.6 ± 0.1, respectively. McCoy [3] also reported the RCR value in epithelial Mt after collagenase dissociation of hamster cheek pouch oral mucosa for succinate to be 2.45, without any reported value for the ADP/O ratio. Our value of 1.65 ± 0.03 for RCR is 73% of that reported by McCoy [3]. 

 In terms of the relationship between metabolism and the function of polymorphonuclear cells (PMNs), it has been established that the chemotactic, phagocytic, and microbicidal activities of these cells are strongly dependent upon their oxidative metabolic process which is driven by Mt [8]. Leukotoxins (Lxs) are a group of exotoxins that produce their primary toxic effects against leukocytes, especially PMNs [18]. Suliman et al. [19] reported that lipopolysaccharide (LPS) exhibited an endotoxic damage on the respiratory function of rat heart Mt. Both commercial Lx and LPS in this study effectively inhibited the respiratory activity of rat gingival Mt (Table 3). This fact suggests that both Lx and LPS may be involved in the attenuated Mt function of inflamed gingival tissues. 

 In conclusion, rat gingival tissue Mt were isolated using the conditions of 50 sec polytron homogenization and 0.120% (w/v) collagenase concentration. The quality of this Mt preparation was confirmed by the demonstrated higher activity of SDH. Furthermore, the quality the Mt preparation was certified by both respiratory parameters such as RCR and ADP/O ratio. Finally, it was found that the RCR of Mt was decreased by both an exotoxin, Lx, and an endotoxin, LPS. 

## Figures and Tables

**Table 1:** Specific activity of succinate dehydrogenase (SDH) in the various fractions in the process of mitochondrial isolation.

Fractions	Specific activity
	(U/mg protein)
S1	0.51 ± 0.05*
S2	0.72 ± 0.05*
S3	0.22 ± 0.02*
N	0.25 ± 0.02*
Mt	5.67 ± 0.32

S1, S2, and S3 designate the first, second, and third supernatant fractions in the process of mitochondrial isolation, respectively, while N and Mt designate the precipitated fractions of the nuclei and mitochondria, respectively, (see Figure 1). Enzymatic digestion was carried out under the conditions of 0.120% (w/v) collagenase for 20 min with 50 sec homogenization. Specific activity of succinate dehydrogenase was measured by the modified Slater method [12].

*Significantly different compared with the mitochondrial (Mt) fraction, *P* < .05.

**Table 2:** Respiratory control ratio (RCR) and adenosine diphosphate/oxygen (ADP/O) ratio in mitochondrial fractions from rat gingival tissues.

Concentration of collagenase [%(w/v)]	Respiratory control ratio	ADP/O ratio
Homogenized for 40 sec		
0.120	1.27 ± 0.04	1.20 ± 0.06
0.125	1.54 ± 0.02	1.39 ± 0.05
0.130	1.43 ± 0.04	1.25 ± 0.02
Homogenized for 50 sec		
0.115	1.45 ± 0.03	1.36 ± 0.03
0.120	1.80 ± 0.05^∗,∗∗ ^	1.65 ± 0.03^∗,∗∗^
0.125	1.39 ± 0.04	1.28 ± 0.03
Homogenized for 60 sec		
0.115	1.50 ± 0.03	1.42 ± 0.02
0.120	1.57 ± 0.03	1.49 ± 0.01
0.125	1.33 ± 0.05	1.23 ± 0.03

The respiratory control ratio (RCR) and adenosine diphosphate/oxygen (ADP/O) ratio of the mitochondrial (Mt) fractions which were obtained by varying the concentration of collagenase and the time of homogenization were measured. After measuring states 1, 3, and 4, the respiratory activity of gingival Mt was assessed by measuring (1) the respiratory control ratio (RCR): the ratio of the respiratory rate (State 3) in the presence of ADP to the rate (State 4) following the expenditure of ADP and (2) adenosine diphosphate/oxygen (ADP/O) ratio: the ratio of ADP removed from the media to the amount of oxygen consumed, according to the Estabrook method [15].

*Significantly different in terms of the highest values between 40 sec and 50 sec homogenizing at *P* < .05.

** Significantly different in terms of the highest values between 50 sec and 60 sec homogenizing at *P* < .05.

**Table 3:** Effects of leukotoxin and lipopolysaccharide on the respiratory control ratio (RCR) in mitochondrial fractions from rat gingival tissues.

	Concentration used	Respiratory control
	(*μ*g/mg Mt protein^a^)	ratio
Control (none)	0.0	1.82 ± 0.05 *R*
Leukotoxin	0.5	1.29 ± 0.05*
	1.0	1.15 ± 0.03*
	2.0	1.05 ± 0.03*
Lipopolysaccharide		
	5.0	1.26 ± 0.03*
	10.0 *a*.	1.13 ± 0.03*
	25.0 *a*.	1.10 ± 0.03*

The respiratory control ratio (RCR) of the mitochondrial (Mt) fractions which was obtained from the conditions of 0.120 % (w/v) collagenase treatment and 50 sec homogenization. At a concentration range of 0.5–2.0 *μ*g/mg Mt protein, leukotoxin was added to a 600 *μ*L of 10 mM HEPES buffer (pH 7.4) containing 0.25 M sucrose and 10 mM KH_2_PO_4_ in the reaction container of an oxygen electrode apparatus, followed by the addition of 100 *μ*g/mL rotenone and 0.6 mM succinic acid. Also, at a concentration range of 5.0–25.0 *μ*g/mg Mt protein, lipopolysaccharide was added in the same manner. RCR was determined polarographically, as shown in Table 2.

^a^Mt protein; mitochondrial protein.

*Significantly different when compared with the control (none) at *P* < .05.
